# Patient-Reported Outcomes After Preoperative Botulinum Toxin A Injection Prior to Abdominal Wall Hernia Surgery: An International Survey

**DOI:** 10.3389/jaws.2025.15523

**Published:** 2025-11-26

**Authors:** Mateusz Zamkowski, Jackie Bullock, Allan Aim, Abdulrahman Alhasso, Marja A. Boermeester, Sara Capoccia Giovannini, Andrew de Beaux, Barbora East, Pilar Hernández-Granados, Miloslav Klugar, Pavlo Kozenko, David Moszkowicz, Sónia Ribas, Sebastian Schaaf, Maciej Śmietański, Mette Willaume Christoffersen, Frederik Berrevoet

**Affiliations:** 1 II Department of Radiology, Medical University of Gdańsk, Gdańsk, Poland; 2 Śmietański Hernia Center, Luxmed Hospital in Gdańsk, Gdańsk, Poland; 3 EHS Patient Advisory Committee, Hernia Patient Support Group, Blackburn, United Kingdom; 4 East Tallinn Central Hospital, Tallinn, Estonia; 5 Spire Murrayfield Hospital, Edinburgh, United Kingdom; 6 Department of Surgery, Amsterdam UMC Location University of Amsterdam, Amsterdam, Netherlands; 7 Amsterdam Gastroenterology Endocrinology and Metabolism, Amsterdam, Netherlands; 8 Department of Surgical Sciences and Integrated Diagnostics, Policlinico San Martino Hospital, University of Genoa, Genova, Italy; 9 Spire Murrayfield Hospital, Edinburgh and Oxford University Hospitals, Oxford, United Kingdom; 10 3rd Department of Surgery of 1st Medical Faculty, Charles University, Motol University Hospital, Prague, Czechia; 11 Abdominal Wall Surgery Unit, Department of General and Digestive Surgery, Fundación Alcorcón University Hospital, Madrid, Spain; 12 Cochrane Czech Republic, Czech Republic: A JBI Centre of Excellence, Czech GRADE Network, Institute of Health Information and Statistics of the Czech Republic, Prague, Czechia; 13 Center of Evidence-Based Education and Arts Therapies: A JBI Affiliated Group, Faculty of Education, Palacký University Olomouc, Olomouc, Czechia; 14 Sambir Rayon General Hospital, Sambir, Ukraine; 15 Université Paris Cité, Service de Chirurgie Digestive, Hôpital Louis Mourier, DMU ESPRIT - GHU AP-HP, Nord - Université Paris Cité, Paris, France; 16 Abdominal Wall Surgery Unit, Department of General Surgery, ULS Póvoa de Varzim-Vila do Conde, Póvoa de Varzim and Hospital Lusíadas Braga, Braga, Portugal; 17 Department of General, Visceral and Thoracic Surgery, German Armed Forces Central Hospital Koblenz, Koblenz, Germany; 18 Zealand University Hopsital Koege, University of Copenhagen, Copenhagen, Denmark; 19 Department of General and HPB Surgery and Liver transplantation, Ghent University Hospital, Gent, Belgium

**Keywords:** botulinum toxin, abdominal wall, hernia, patient-reported outcomes, prehabilitation

## Abstract

**Introduction:**

Botulinum Toxin A is increasingly used as a preoperative adjunct in the management of complex abdominal wall hernias, particularly in those with wider defects and/or loss of domain. While its anatomical and surgical benefits have been documented, patient-reported outcomes remain underexplored.

**Methods:**

An international, retrospective, observational study was conducted using a structured, anonymised survey available in five languages (German, English, Polish, French, Spanish). The survey included nine closed-ended and one open-ended question assessing pain perception, mobility, respiratory, gastrointestinal, and urinary function, and changes in abdominal contour. It was distributed by medical teams and through patient support forums between 2024 and 2025. Patients included had received BTA injections 4–6 weeks prior to elective hernia surgery.

**Results:**

Seventy patients from multiple European centres completed the survey. Pain during injection was minimal in 71.5% of cases, with 85.7% reporting complete resolution of pain within 1–3 days. Most respondents (74.3%) experienced no breathing difficulties and only mild symptoms in 18.6%. Mobility remained unchanged in 80%, while 15.7% noted slight deterioration. Changes in urinary and bowel function were uncommon and mostly transient. Over half of patients reported visible changes in abdominal shape. No severe complications were identified.

**Conclusion:**

This international patient survey suggests that BTA injections as preoperative preparation for complex hernias is well tolerated, with limited perceived side effects and functional disruption. These findings support its continued use and prompts further prospective data collection.

## Introduction

The use of Botulinum Toxin A (BTA) has gained popularity as a preoperative adjunct in the management of complex abdominal wall hernias, especially those associated with loss of domain (LOD) [1, 2]. By inducing temporary chemical denervation of the lateral abdominal wall muscles—external oblique, internal oblique, and transversus abdominis—BTA facilitates muscle elongation and expansion of the abdominal cavity, potentially enabling tension-free fascial closure [3].

Multiple studies have demonstrated anatomical and biomechanical benefits of BTA, including reduced fascial tension and increased intra-abdominal volume and a decrease in the intra-abdominal pressure [4, 5]. As a result, BTA is now employed in many high-volume centres and hernia programmes worldwide, though protocols vary in terms of drug formulation, dilutions, dosage, and injection technique.

Despite increased clinical use, patient-reported outcomes related to BTA remain underexplored. Most publications focus on imaging-based or surgical endpoints, with limited attention paid to the subjective experiences of those receiving the injections [6–11]. Understanding how patients perceive pain, mobility, breathing, and general tolerability is important, particularly as BTA becomes more routinely and widely used in abdominal wall repair surgery.

Moreover, while BTA is generally considered safe, rare complications have been recently reported—most notably transient respiratory compromise in older adults or frail patients [12]. Spirometric studies have attempted to assess these risks more objectively, but definitive conclusions are still lacking [13].

This international, patient-reported survey was designed to address this knowledge gap. By collecting subjective experiences from diverse populations and clinical settings, the study aims to assess the tolerability and perceived side effects of BTA during the preoperative phase of complex hernia treatment.

## Materials and Methods

This was a retrospective observational study based on a structured, anonymized patient survey evaluating subjective tolerance and perceived effects of preoperative BTA administration in the context of complex abdominal wall hernia repair. The study was based on a multinational, retrospective, observational survey conducted among patients who had received BTA as part of prehabilitation for complex abdominal wall hernia repair between 2024 and 2025. Contributions were collected from multiple centres across Europe. Participating centres included institutions in Germany, United Kingdom, Poland, France and Spain. Patients were recruited either through direct clinical contact or via hernia-focused patient groups, depending on the centre.

### Patient Selection

Patients were eligible for inclusion if they had received BTA injections to the lateral abdominal wall muscles (external oblique, internal oblique, and transversus abdominis) prior to scheduled elective hernia repair and had completed a minimum of 4 weeks of follow-up after injection. Only patients with completed abdominal wall repair were included to ensure they could report on the full perioperative experience. All participants received BTA in preparation for large midline or parastomal hernias with or without loss of domain. The survey was intentionally designed as fully anonymized; therefore, no identifying or demographic information such as age, gender, or detailed medical history was collected. Likewise, information regarding the psychological background or chronic pain status of the participants was not requested to ensure compliance with data protection regulations. This anonymity limited the possibility of subgroup analyses based on demographic or clinical characteristics but allowed for broad international participation.

### Survey Design and Distribution

A structured questionnaire was developed in five language versions (German, English, Polish, French, Spanish) to accommodate the international patient population. The initial version was created by the study authors based on expert consensus within the European Hernia Society Guidelines Group. Key domains—such as pain perception, functional limitations, and organ-specific effects—were identified through clinical experience and a review of previously published studies on BTA in abdominal wall surgery. The survey included 9 single-choice questions using numerical or descriptive scales and one open-ended question (Supplementary Material 1). Translations were carried out by native speakers with a clinical background. Although the questionnaire was not formally validated through pilot testing or interview-based psychometric assessment, it was pragmatically designed for quick and simple application and international use. Topics addressed included pain during and after injection, perceived changes in mobility, breathing, bowel and urinary function, and subjective abdominal wall morphology. Importantly, patients were actively involved in the development of the questionnaire, providing feedback on clarity, relevance, and comprehensibility of the items. Their participation ensured that the survey addressed issues most meaningful from the patient perspective and enhanced the overall validity and accessibility of the instrument. Respondents were invited to complete the questionnaire at least 4 weeks after BTA injection and within 2 weeks after surgery. However, exact timing was not uniformly recorded, which may have introduced recall bias.

### BTA Administration Protocol

Patients received Botulinum Toxin A (e.g., Botox®, Dysport® or Xeomin®), reconstituted in saline and administered under ultrasound guidance into 2, 3 or 5 injection points on each side along the anterior axillary line. Doses and volume distribution varied depending on the surgeon or radiologist performing the procedure. All injections targeted the lateral abdominal wall musculature, and patients were instructed to avoid strenuous activity for 48 h post-procedure. Information on injection technique, number of punctures, drug formulation, and use of anesthesia varied among centers and was not consistently recorded, precluding formal subgroup analysis by technique or operator. The heterogeneity of administration technique was noted and considered a limitation of the study.

### Data Analysis

Responses were compiled and analysed using Microsoft Excel®. Descriptive statistics were used to present the distribution of responses for each question. No inferential statistical testing was performed due to the observational nature and heterogeneity of the cohort. Results are presented as proportions and frequency tables. Due to missing demographic and procedural data (e.g., number of punctures, anesthesia type, or operator specialty), stratified comparisons could not be conducted, and results were therefore summarized as descriptive statistics.

## Results

A total of 70 complete responses were collected from patients in five European countries: Germany (n = 24), the United Kingdom (n = 19), Poland (n = 14), France (n = 11), and Spain (n = 2). The survey assessed pain perception, functional symptoms, and potential side effects (Table 1) at various time points following BTA administration to the lateral abdominal wall muscles.

**TABLE 1:** Detailed survey response distribution.

Question	Response options	Germany n = 24	UK n = 19	Poland n = 14	France n = 11	Spain n = 2	Overall n = 70
Pain during injection (0–10)	0–2 3–5 6–8 9–10	5 14 5 0	10 1 7 1	8 4 2 0	3 4 2 2	1 0 1 0	27 (38.6%) 23 (32.9%) 17 (24.3%) 3 (4.3%)
Pain on day after injection (0–10)	0–2 3–5 6–8 9–10	20 3 1 0	14 3 1 1	10 4 0 0	10 1 0 0	1 1 0 0	55 (78.6%) 12 (17.1%) 2 (2.9%) 1 (1.4%)
Pain 1 week after injection (0–10)	0–2 3–5 6–8 9–10	21 3 0 0	16 0 2 1	14 0 0 0	9 2 0 0	0 1 1 0	60 (85.7%) 6 (8.6%) 3 (4.3%) 1 (1.4%)
Duration of pain	1–3 days 1 week 2 weeks 3 weeks 1 month 2 months Still present on day of surgery	22 2 0 0 0 0 0	14 1 1 0 0 2 1	14 0 0 0 0 0 0	9 1 0 0 1 0 0	1 0 0 0 1 0 0	60 (85.7%) 4 (5.7%) 1 (1.4%) 0 2 (2.9%) 2 (2.9%) 1 (1.4%)
Breathing difficulty	No Mild Moderate Severe	21 2 1 0	12 5 1 1	9 4 1 0	8 2 1 0	2 0 0 0	52 (74.3%) 13 (18.6%) 4 (5.7%) 1 (1.4%)
Change in abdominal shape	None Small Large	15 8 1	5 9 5	2 10 2	7 4 0	1 1 0	30 (42.9%) 32 (45.7%) 8 (11.4%)
Difficulty in bed/chair movement	Improvement None Slightly worse Significantly worse	1 21 2 0	0 14 5 0	0 11 3 0	2 8 1 0	0 2 0 0	3 (4.3%) 56 (80%) 11 (15.7%) 0
Changes in bowel movements	Improvement None Slightly worse Significantly worse	0 22 2 0	2 13 4 0	0 14 0 0	2 8 0 1	0 1 1 0	4 (5.7%) 58 (82.9%) 7 (10%) 1 (1.4%)
Changes in urination	Improvement None Slightly worse Significantly worse	1 23 0 0	2 16 1 0	0 14 0 0	1 10 0 0	0 2 0 0	4 (5.7%) 65 (92.9%) 1 (1.4%) 0

### Pain and Discomfort

Pain during injection was generally low: 27 patients (38.6%) rated it 0–2/10, 23 (32.9%) rated it 3–5, and only 3 patients (4.3%) reported severe pain [9, 10].

Pain on the day after injection was minimal for most participants, with 55 patients (78.6%) rating it 0–2/10 and only 1 patient (1.4%) reporting severe pain. At 1 week post-injection, 60 patients (85.7%) continued to report minimal or no pain.

Pain duration was short in the majority: 60 patients (85.7%) reported resolution within 1–3 days. Only 1 patient (1.4%) still experienced pain on the day of surgery.

### Respiratory and Abdominal Symptoms

Breathing difficulty was absent in 52 patients (74.3%), mild in 13 (18.6%), moderate in 4 (5.7%), and severe in 1 case (1.4%).

Changes in abdominal contour were reported by 40 patients (57.1%): 32 (45.7%) described a small change and 8 (11.4%) a large change.

### Mobility and Gastrointestinal/Urinary Effects

Regarding bed or chair mobility, 56 patients (80%) reported no change, 11 (15.7%) noted slight worsening, and 3 (4.3%) reported improvement.

Changes in bowel movements were infrequent: 58 patients (82.9%) reported no change, 7 (10%) slight worsening, and 4 (5.7%) improvement.

Changes in urinary function were minimal: 65 patients (92.9%) reported no change, 4 (5.7%) reported improvement, and 1 (1.4%) slight worsening.

### Summary Table

All responses with numerical distributions are presented in Table 1. The full questionnaire is provided as (Supplementary Material 1).

## Discussion

This patient-centred survey highlights the tolerability and low prevalence of potential side effects following BTA pretreatment in hernia surgery. Pain levels associated with injection were generally low and short-term. Most patients experienced no adverse changes in function, and a substantial proportion reported improved abdominal compliance, consistent with clinical goals of BTA. Mild respiratory symptoms were reported in a minority of patients but did not interfere with daily activities. These findings support the safety of BTA from the patient perspective and underscore the importance of appropriate counselling and post-injection support.

In recent years, there has been a growing number of studies emphasising the clinical advantages of BTA in the management of complex abdominal wall hernias [6, 8, 9, 14–16]. While clinical studies highlight various surgical advantages of BTA, including improved fascial closure rates, these outcomes fall outside the scope of the present analysis. This survey focused specifically on patient-reported tolerance and functional perceptions of BTA in the preoperative period.

The number of peer-reviewed articles focusing on BTA in abdominal surgery is rapidly increasing, reflecting exponential increased interest and expanding clinical experience. Continued evolution of injection practices reflects growing clinical interest, yet patient-reported experiences remain a critical perspective in evaluating the tolerability and real-world implications of such protocols [17]. Reports also highlight the possibility of adverse effects. Rare but significant cardiopulmonary complications—such as transient respiratory insufficiency or cardiovascular instability—have been described, particularly in older adults or comorbid patients [12]. The authors advise to consider the cardiopulmonary history and neurological disorders that could result in decreased respiratory function, pulmonary functional capacity, and anaerobic threshold, to prevent the potentially serious adverse effects of BTA. Pulmonary function tests, like spirometry (in pulmonary comorbid patients) and Cardiopulmonary Exercise Testing (in cardiopulmonary comorbid patients), could give a good indication whether complications following chemical BTA are predictable. These concerns are further supported by anecdotal accounts and case series, underlining the importance of careful patient selection, clinical monitoring, and individualised risk–benefit assessment when considering BTA administration. Such concerns have prompted attempts to objectively assess respiratory function following BTA injection. In one observational study, spirometric testing before and after administration did not demonstrate significant deterioration in pulmonary parameters. However, the results remained inconclusive and did not provide definitive evidence for or against a causal relationship between BTA use and respiratory compromise [13].

While the available data suggest that BTA is well tolerated in the vast majority of cases, the absence of high-quality, controlled studies reinforces the need for cautious use in vulnerable populations and highlights the value of real-world, patient-reported data to guide future clinical protocols.

### Subjective Nature of Pain Perception

Pain is inherently subjective and influenced by multiple factors, including psychological state, prior experiences, cultural background, and individual pain thresholds. As shown in various studies, identical stimuli can be rated very differently by different individuals [18]. In our cohort, the majority of patients reported low pain scores during injection (71.5% rated 0–5/10), and discomfort typically resolved within 1–3 days in nearly 80% of cases. Nevertheless, a small minority reported high pain scores, including one patient rating the injection as 10/10. Such isolated responses should not automatically be interpreted as procedural complications, but rather as part of the expected variability in pain perception. These findings highlight the importance of pre-procedure counselling and cautious interpretation of patient-reported outcomes in studies of this nature.

### Generalisation/Applicability

The survey also reveals that while changes in abdominal wall shape were common, they were rarely distressing or functionally limiting. The frequency of reported changes in abdominal wall contour—typically described as minor and non-distressing—highlights the value of informing patients about expected, benign changes during preoperative counselling.

This study provides patient-centred data on the tolerability of BTA in hernia prehabilitation, highlighting its generally high tolerability among patients. As BTA becomes more integrated into surgical protocols, incorporating subjective experiences into routine evaluation will help tailor patient selection, improve shared decision-making, and balance perceived benefits with emerging safety concerns [19].

To further enhance patient-centred care, we propose that this questionnaire be routinely implemented in clinical practice as a standard tool for patients undergoing preoperative Botulinum Toxin A injection. Regular collection of such data at the local level would allow clinicians to monitor patient perspectives systematically, identify unexpected tolerability issues, and refine counselling strategies. Incorporating standardised patient-reported outcomes into routine care may also support benchmarking across institutions and contribute to the development of evidence-based guidelines.

### Limitations

This study has several important limitations that should be acknowledged.

First, the questionnaire used in this survey was not formally validated. It was designed pragmatically to ensure accessibility across languages and clinical contexts, but it lacks psychometric validation or pilot testing, which may influence reproducibility and generalizability of the findings.

Second, demographic and clinical background data—including patients’ age, gender, medical history, psychological status, history of chronic pain, and use of analgesics—were not collected in this anonymized survey. These factors are known to significantly influence pain perception and could have contributed to variability in the reported outcomes.

Third, there was notable heterogeneity in procedural details, including the number of puncture sites, doses and dilution of Botulinum Toxin A, as well as operator specialty (radiologist vs. surgeon) and use of anesthesia or sedation. Because these variables were not consistently recorded across participating centers, they could not be analyzed in relation to pain intensity or tolerance. It is possible that patients who underwent the procedure without anesthesia or sedation may have reported higher discomfort, although this could not be formally assessed. Future prospective studies should capture and compare these variables systematically.

Fourth, the international cohort was unevenly distributed, with variable patient numbers across countries (for example, 24 participants from Germany versus only 2 from Spain). Cultural and linguistic differences may have influenced subjective interpretation of pain or functional symptoms, potentially introducing reporting bias.

Finally, because participation was voluntary, patients who experienced serious complications may have been less likely to respond, introducing potential survivorship bias. Future prospective trials, such as the ongoing RCT NCT06499324 in France, are expected to provide structured data on BTA use under standardized protocols.

It is also important to acknowledge that patients who may have experienced severe complications following BTA administration—such as cardiopulmonary deterioration—are unlikely to have responded to the survey, introducing a potential survivorship bias. Given the relatively small sample size, this study is not powered to detect rare or serious adverse events. Future research efforts should include registry-based data collection to better define the true incidence of uncommon but potentially serious complications associated with BTA administration.

## Conclusions

This study demonstrates that preoperative BTA injections are generally safe and well tolerated by patients undergoing preparation for complex ventral hernia repair. The majority of respondents reported minimal pain, few functional complaints, and no significant gastrointestinal or urinary disturbances. Mild respiratory symptoms were infrequent and did not interfere with daily activities.

These patient-reported findings provide reassurance regarding the subjective tolerability of BTA and support its role as a patient-acceptable component of prehabilitation protocols. By focusing on the patient experience, the study contributes with a valuable dimension to ongoing evaluation of BTA in abdominal wall surgery.

Future prospective studies should aim to validate these findings in larger, more diverse populations and assess long-term outcomes, including functional recovery and health-related quality of life.

## Significance of the Study

This study represents one of the first international efforts to systematically evaluate patient-reported outcomes following preoperative BTA injection in abdominal wall hernia repair. These results offer meaningful insight into patient tolerance, perceived side effects, and functional safety—elements that are essential for informed consent, shared decision-making, and future guideline development. By centring patient experiences, this research adds a valuable dimension to the growing body of evidence supporting BTA in hernia surgery.

## Data Availability

The original contributions presented in the study are included in the article/Supplementary Material, further inquiries can be directed to the corresponding author.
