# Supplementary material for: Patient-Reported Outcomes After Preoperative Botulinum Toxin A Injection Prior to Abdominal Wall Hernia Surgery: An International Survey
Source: J Abdom Wall Surg. 2025 Nov 26;4:15523. doi: 10.3389/jaws.2025.15523 (PMC12689444; doi:10.3389/jaws.2025.15523)
Supplement: Supplementary file 1 [file DataSheet1.pdf]

Full Patient Questionnaire

1. How painful was the injection procedure (0 = none, 10 = worst pain ever experienced)?

2. How would you rate the pain on the day after injection? (0 = none, 10 = worst pain ever experienced)

3. How would you rate the pain 1 week after the injection? (0 = none, 10 = worst pain ever experienced)

4. How long did the injection site hurt after the procedure?

   ☐ 1-3 days ☐ 1 week ☐ 2 weeks ☐ 3 weeks ☐ 1 month
   ☐ 2 months ☐ still present on day of surgery

5. Did you feel any breathing difficulties or chest pressure after the injection?

   ☐ In the days and weeks following your injection of Botulinum toxin A and up to the day of your surgery did you notice any changes in your breathing
   ☐ No change
   ☐ I felt a bit more breathless but I was still able to go about me normal activities
   ☐ I felt a lot more breathless than normal and this reduced my ability to go about my normal daily activities
   ☐ I felt very breathless and this limited my ability to go about my normal activities

6. Did you notice any visible change in the shape of your abdomen?

   ☐ There was no change in the shape of my tummy.
   ☐ I noticed a small change in the shape of my tummy.
   ☐ I noticed a big change in the shape of my tummy.

7. Did you feel any difficulties in bed movement (turning, sitting up)?

   ☐ I found it easier to get in and out of bed or to stand up from my chair
   ☐ I didn't notice any difference in getting in or out of bed or standing after being sat down
   ☐ I felt it was a little more difficult to get in and out of bed or to stand after being sat down
   ☐ I found it a lot harder to get in and out of bed or to stand after being seated

8. Did you notice any change in urination or bowel movements?

☐ I found it easier to have bowel movements after the injection of botulinum toxin A
☐ I didn't experience any changes in my ability to have bowel movements following my injection of botulinum toxin
☐ I found it a little more difficult to have bowel movements following my injection of botulinum toxin
☐ I found it extremely difficult to have bowel movements following my injection of botulinum toxin A

9. In the days and weeks following your injection of botulinum up until your surgery did you notice any difference in your ability to pass urine?

☐ I found it easier to pass urine after my injection of botulinum toxin A
☐ I found no difference in my ability to pass urine following my injection of botulinum toxin A
☐ I found it a little bit more difficult to pass urine after my injection of Botulinum toxin
☐ I found it a lot more difficult to pass urine after my injection of Botulinum toxin A

Additional Comments regarding BTA injection procedure:
